# “We also communicate through a book in the diaper bag”—Separated parents´ ways to coparent and promote adaptation of their 1-4 year olds in equal joint physical custody

**DOI:** 10.1371/journal.pone.0214913

**Published:** 2019-04-10

**Authors:** Malin Bergström, Anna Sarkadi, Anders Hjern, Emma Fransson

**Affiliations:** 1 Department of Public Health Sciences, Centre for Health Equity Studies (CHESS), Stockholm University/ Karolinska Institutet, Stockholm, Sweden; 2 Clinical Epidemiology, Department of Medicine, Karolinska Institutet, Stockholm,Sweden; 3 Department of Public Health and Caring Sciences, Uppsala University, Uppsala, Sweden; Public Library of Science, UNITED KINGDOM

## Abstract

Joint physical custody (JPC) refers to a practice where children with separated parents share their time between the parents’ respective homes. Studies on parents’ views of JPC for young children are scarce. The aim of this interview study was to explore parents’ perceptions on how they experience and practice equally shared JPC for their 1–4 year-olds in Sweden. Forty-six parents (18 fathers and 28 mothers) of 50 children (31 boys and 19 girls) under 5 years of age were interviewed. Parents were recruited through information in the media and represented a broad range of socioeconomic backgrounds, as well as both voluntary and court-ordered custody arrangements. The interviews were semi-structured and analyzed using systematic text condensation. Two themes emerged regarding the research question. In the first theme, *Always free*, *never free*, parents described their striving to coparent without a love relationship. While they appreciated the freedom of being a “half-time parent”, doing things one’s own way, they felt constrained by the long-term commitment to live close to and keep discussing child rearing issues with the ex-partner. Good communication was key and lessened parent’s feelings of being cut-off from half of the child’s life. When JPC was ordered by court or conflicts were intense, parents tried to have less contact and worried when the children were in the other home. The second theme, *Is it right*, *is it good*?, included descriptions of how the parents monitored the child’s responses to the living arrangement and made changes to optimize their adjustment. Adaptations included visits for the child with the other parent mid-week, shared meals or adapting schedules. In conclusion, these parents worked hard to make JPC work and cause minimal damage to their children. Most parents were pleased with the arrangements with the notable exception of couples experiencing ongoing conflict.

## Introduction

Joint physical custody (JPC) refers to a practice where children with separated parents share their time between the parents’ respective homes. According to recent data nearly 50 percent of Swedish preschoolers with separated parents have equal JPC (50/50 shares) and another 28 percent live with both parents but on an unequal basis [1]. Also in other Western countries JPC is increasing and concerns between 20 and 25 percent of children with separated parents in countries such as Belgium, The Netherlands, Denmark, Norway and certain states in the US [2–10]. More gender equal parental roles, fathers’ engagement in parenting and women’s participation in the labor force are plausible reasons behind the popularity of this practice among separated families [11]. The increase of children who share their time between two homes can be argued to be one of the biggest changes in children’s living conditions in recent years.

In international studies JPC may refer to children living 35–65% with each parent [12]. Since JPC is more common in Sweden than elsewhere, and in particular for the youngest age groups, Swedish studies often apply a more narrow categorization of the practice than international studies: e*qual or almost equal time with each parent*, and often include a category of children living *mostly* with one parent beside the categories nuclear families, JPC and single care families [1].

How parents organize their responsibilities after a separation and decide on custody and their children’s living arrangements for has been found to be related to qualitative as well as socioeconomic family factors [3]. One such qualitative factor is parents’ coparenting relationship, i.e., their ability to coordinate their parental roles, communicate, agree and support each other, has been shown to both affect and predict the degree of a father’s involvement in parenting [13,14,15]. This involvement is, in turn, related to parents’ subsequent conflict levels [16]. Norwegian researchers have found that coparenting quality has larger impact on parents’ choice of living arrangement for their children than their educational level [3,17].

Gender‐equal practices have been identified as strong predictors for JPC, partly because mothers in more equal relationships tend to trust the father’s caring capacity and thus are inclined to continue to share parenting responsibilities after the separation [17]. Sweden’s history of gender-neutral regulations for parental leave during the child’s first years, and family polices that have explicitly strived to increase gender equality among parents, may explain Swedish parents’ inclination to share the care of their children after a divorce [18]. There are, however, researchers who argue that families who practice JPC do not differ from those with single mother care in terms of conflict or parental communication after the divorce [19]. It does however seem likely that early involvement in parenting by both parents, such as sharing the parental leave during the child’s first year, will influence subsequent parent-child relations and the mutual coparenting trust and confidence between the parents, particularly after parental separation.

Father involvement in parenting is per se established as an important predictor of children’s positive development, health and behavior [20]. The relationship between the parents also influences parent-child relationships and high coparenting quality has been shown to contribute to a positive emotional family climate and to affect child mental health and social adjustment positively [21,22]. The quality of the coparenting relationship has in fact been shown to be closer linked to child outcomes than other aspects of parents’ relationship, such as intimate and love-related aspects [15,23]. Particularly after a parental divorce coparenting has been described as a key mechanism for how children fare mentally [24,25]. Some authors argue that the psychological functioning of children after their parents’ marital dissolution is not associated with the end of the marital relationship itself, but with the family functioning after this transition [26,27]. It has been suggested that coparenting quality in separated parents’ relations rely on their ability to separate a past romantic relationship from the coparental relationship that needs to be developed and expanded [28]. Coleman et al. (2014) found that parents who could establish new roles for themselves and their coparents were more likely to create a resilient coparenting relation that could develop over time [29]. Successful transitioning of the relationship required changes in how parents thought and felt about their coparents but also behavioral changes such as avoiding conflict and keeping communication neutral with a child focus. One mother quoted in their study said “Picture perfect? No. One hundred percent of the time? No. Do I want to have lunch with [my ex-husband] every day? No. Would I on Tuesday if we needed to talk about my child? Absolutely”. The establishment of an effective coparenting relation has been argued to be an active commitment by the parents and couples who trust each other’s care giving capacities will be more inclined to work on such relationship [28,30]. However, how parent-child relations are affected by the parental relationship, in particular after a divorce, is not fully elucidated. In contrast to the findings of the studies referenced above, the quality of the individual parent-child relationship was argued to be more important for child wellbeing than parents’ conflicts and abilities to coparent in a recent American study [31].

Despite the positive consequences for child health and wellbeing of father involvement in parenting, JPC has been hypothesized to expose children to stress from the hassles of frequent moves, two family cultures [32] and feelings of being torn between parents [33]. For young children, experts have expressed concerns about how children’s attachment relations and subsequent development is affected by the frequent separations from the mother, imposed by JPC [34]. Attachment theory describes how children form relationship(s) of a special emotional quality from birth onwards and the quality of these early attachment patterns has been found to predict developmental outcomes later in childhood [35]. Concerns have been raised regarding the risks associated with frequent separations from the primary attachment figure (i.e the mother) and some experts are cautious about overnights during the first years [34]. Other scholars instead recognize children’s capacity to establish parallel attachment relationships and recommend overnights in order to strengthen relations to both parents [36, 37]. That a father independently cares for the child is, by some experts, in fact regarded a prerequisite for evolvement of the child’s attachment to him [38]. Lack of attachment to the father is hypothesized to leave the child with a more vulnerable emotional environment, since the attachment relation to the mother may not be secure [39].

Several studies have shown better child-father relations in JPC compared with single mother custody [40–42]. For school children and teenagers also child health and behavioral adjustment have been shown to be better in JPC, compared with living with only one of the parents [41–43]. In a previous publication from our research group we showed that Swedish 3–5 year olds in equal JPC had less psychological symptoms than their peers who lived more or only with one parent according to parents and preschool teachers [1]. In parental reports, children in JPC and those in intact nuclear families had similar outcomes, while the preschool teachers reported lower unadjusted symptom scores for children in intact nuclear families. This study was conducted in a sample of parents with relatively high education and there was no information on e.g. coparenting quality. For younger children, 0–2, the empirical data is scarce and since equal JPC, outside of Sweden, is uncommon among infants and toddlers, the international literature has focused on child outcomes in relations to *overnights* with the second parent (mainly the father), rather than JPC. Studies have indicated less secure attachments relation to mothers among children with such overnight visits, especially for infants [44–46]. Attachment quality in relation to the fathers has not been assessed. Findings on health and behavioral outcomes in infants and toddlers with vs. without overnights with the fathers point in different directions [46–49]. Authors of some of these studies have commented on the influence of the parental relationship on the child outcomes, such as mothers’ trust in the fathers’ parenting capability, satisfaction with the coparenting relation [46] as well as parents’ communication and conflict levels [50]. In a follow-up study of one of these studies the author found that mothers’ positive attitudes to fathers’ engagement in parenting contributed to explain the benefits of the overnights for the children [49]. Also the fact that high proportions of the parents in the included families had never lived together has been proposed to contribute to subsequent coparenting problems and to problems for the children to establish attachment bonds to their fathers [42,45–47,50,51]. In conclusion, the literature on how infants and toddlers fare in JPC is scarce and contradictory. Available studies are also weighed by methodological problems, such as measurement issues and sample bias. In addition, societal context is highly relevant for this practice, where opting for JPC might be the norm in some countries, but an unusual practice in others, affecting both researchers and research subjects. In a Norwegian survey by Skjørten et al (2007), in a country where JPC is common, 92% of separated parents with JPC arrangements reported good parental cooperation and the majority reported having frequent contact with the other parent, at least every week [52].

Research on parents’ own experiences of JPC may have important additions to the literature when we try to understand how they negotiate coparenting and how they try to assist their children’s adaptation in a JPC situation. In a previous publication we showed that, among a sample of Swedish parents, involved fatherhood was an ideal goal and JPC was considered ‘a given’ since it was assumed to be in the best interest of the child [53]. How coparenting is described and organized for postdivorce families with very young children and in JPC arrangements is scarcely, if at all, described in the literature.

## Aim

The aim of this interview study was to explore parents’ perceptions on how they experience and practice equally shared JPC for their 1–4 year-olds.

## Methods

### Recruitment

Interested parents were recruited through advertisements in Swedish newspapers, radio and TV from December 2011 to February 2013. Participants were invited to fill out a form on the research group’s website. To include parents of diverse backgrounds and experiences we advertised in local as well as in national media and continued recruitment until we had reached saturation, i.e. no new information was obtained in additional interviews conducted. Inclusion criteria was currently having a child 0–4 years of age in JPC. We aimed to reach parents with different backgrounds, experiences and reasons for JPC. JPC was defined as the child living about equal amounts of time in each parent’s home. All parents who fulfilled the inclusion criteria were contacted by phone or e-mail and given oral and/or written information about the study. All parents who were contacted choose to participate and were included in the study. The present study is part of a larger qualitative project to elucidate different aspects of parents’ experiences of JPC with young children. The study was approved by the Ethics committee at Karolinska Institutet, Dnr 2011/1493-31/5 and all procedures were in accordance with the Helsinki declaration.

### Participants

We interviewed forty-six parents (28 mothers and 18 fathers) of 50 children (31 boys and 19 girls). All children were under 5 years of age and in JPC arrangements. For one child both the mother and the father participated with individual interviews. For the other children only one parent participated in the study. The mean age for children at the time of parental separation was 21 months, ranging from 0–49 months and mean time from separation was 16 months. Two parents had split up from their partner already during pregnancy and two during the first months after birth. Descriptive statistics were computed for numeric data and are presented in Table 1.

**Table 1:** Parents’ reasons for JPC and sociodemographic characteristics of parents and children presented as mean values, ranges and standard deviations (SDs) or as numbers and percentages.

	Children (n = 50)	Fathers (n = 18)	Mothers (n = 28)
***Child variables***			
Child age in months, mean (range; SD)	37 (13–59; 10.4)		
Child age in months at parental separation, Mean (range; SD)	21 (0–49; 11.4)		
Child gender, girl n, (%)	19 (38)		
***Parental variables***			
Age in years, mean, (range; SD)		36 (27–50; 6.0)	34 (26–44; 4.3)
Older children (>5 years), n (%)		3 (17)	8 (29)
New partner, n (%)		8 (44)	9 (32)
*Highest level of education*, *n (%)* Primary school Secondary school College or University Missing		1 (6) 4 (21) 12 (67) 1 (6)	1 (4) 2 (7) 25 (89)
Monthly Income* n (%) Low (<13.500) Median (13.500–31.500) High (>31.500) Missing		1 (6) 4 (21) 10 (56) 3 (17)	0 16 (58) 6 (21) 6 (21)
*Reasons for JPC*			
Mutual agreement, n (%)		11 (62)	23 (82)
Mediation, n (%)		4 (21)	3 (11)
Court decision, n (%)		3 (17)	2 (7)

* Low and High income represents the lowest and highest income quartiles in Sweden 2013 (Statistics Sweden, 2013).

### Interviews

Parents were re-informed verbally about the aim of the study and the possibility to withdraw from participation at any time-point, before commencement of the telephone interview. Their oral consent to participate and to record the interview was recorded. We used a semi structured interview guide covering various themes but allowing for follow-up questions and the interviews lasted between 30 and 120 minutes. The interviews covered themes concerning the parents' attitudes and experiences of JPC for themselves and their children. The interview guide was pilot tested with one parent (a mother) and no alterations to the guide was made after the pilot telephone interview. This interview was hence included in the sample. For the purpose of this article we used descriptions and answers relating to parental experiences of practicing JPC, whereas parental attitudes to choosing JPC have been reported elsewhere [53].

### Analysis

Background data were collected from the interviews and from the web form where the parents had entered their interest to participate and their background data. Analysis of the interview data was performed using systematic text condensation [54]. First, all interviews were read and re-read by all authors to obtain a comprehensive picture of the data as a whole. Then, recurrent themes reflecting the parents’ experiences and thoughts regarding how the JPC practice was performed and experienced were identified. Meaning units were thereafter identified and grouped on the basis of identified themes, after which the units in each theme were sorted into categories describing different aspects of the theme. Once a category was assigned, the condensation process followed, where the content of each category was summarized in 2–3 sentences as if it were expressed by a single participant, hence integrating all statements in that category into a single statement (Table 2). After condensation, the analytical text was formulated and quotes were selected from the units assigned to that category to exemplify the contents. The selected quotes were labelled with the respective interview number and the sex of the parent. All quotes are verbatim transcriptions of the interviewee’s words; parentheses (…) indicate that some of the text has been omitted due to space restrictions. Finally, the resulting themes and categories were compared with the unbroken original interview texts (re-contextualization) to ensure that they fit the data. To achieve dependability of the analytical process, the identification of themes was conducted separately by the three co-authors who then met to discuss and agree on the final themes. The categorization process was done individually with each author responsible for separate themes.

**Table 2:** The steps of the analysis process in systematic text condensation according to Malterud, 2012.

Steps in data analysis	Examples from the data
1. Total impression of the data:	Despite not having a partner relation anymore, parenting was often still regarded as a common task. Cooperation was seen as necessary due to practical reasons but also for the child’s adaptation and wellbeing. For the vast majority the amount of contact was intense, with several updates weekly.
*→ Finding Themes*	Besides the practical reasons this also lessened the parent’s feelings of being cut-off from half the child’s life. Many also spoke about the new liberty of being a single parent, not having to compromise, while they felt obliged to always stay in contact and probably live close to their ex-partner. Those with high conflict worried more and wished they had single care of their child.
	Theme: ALWAYS FREE, NEVER FREE
2. Identifying and sorting relevant text units under the designated theme	*”(sharing the responsibilities) worked well before and it is nice that it can be transferred also to this type of relationship*!*”* Father to 18 months old boy *”We communicate well*, *we help each other and strive to continue to share*, *not only the practical*, *but also in fact the positive parts*.*”* Mother to 3 year old boy
*→ from Themes to Categories*	*“The love relationship between me and X is gone*. *But our common parenthood is as important as always*. *We strive to bridge the fact that we are away from [the child] half the time*.*”* Mother to 18 months and 3 year old girls A goal and a challenge for some of the parents was to transform the prior love relation to a common and lasting relation as parents. They worked to support each other and share both the positive and negative aspects of parenting.
	Category: Common parenthood without a love relation
3. Condense the meaning in each category as if it were a story told by a parent	I see a parenting relation with good communication as a prerequisite for my child’s adaptation in this living arrangement. For us this includes common bank accounts and birthday parties. But to support each other on practical issues is not enough.
*→ from Category to meaning through abstraction*	Our ability to communicate is necessary to help our kids through the separation and to make them feel secure in joint physical custody.
4. Summarize each Category, prepare the analytical text and select quotes	A goal and a challenge for some of the parents was to transform the prior love relation to a common and lasting relationship as only parents. They worked to support each other and share both the positive and negative aspects of parenting.
*→ From abstraction to presentation*	
5. Compare the resulting themes and categories with the unbroken text	The theme and one or more of its categories appear in every re-read interview.
*→ Recontextualisation*	

## Results

Parents reported a large variation of how they practiced JPC, in terms of the child’s schedule with the parents. For 21 (42 percent) of the children the parents had full week schedules or other long periods (such as 8+6 days). Two families described having a “booster” gathering in the middle of the period, which implied an extra meeting for the child with a parent during the other parent’s week. The parent could, for example, pick the child up from the preschool and spend the afternoon together or have the child for a one night sleepover. Ten children (20 percent) moved twice a week (4+3 or 5+2 days), 19 (38 percent) moved 3 times a week or more often (for example 2+2+3 or moving every other or every day).

Two themes emerged in response to the question on how parents experience and practice equally shared JPC with children 1–4 years of age, Table 3. In the theme *Always free*, *never free* parents described their strivings to coparent without a romantic relationship. Many also spoke about the new liberty of being a single parent, not having to compromise, while they felt obliged to always stay in contact and probably live close to their ex-partner. Those with high conflict worried more and wished they had single care of their child. The theme *Is it right*, *is it good*? includes descriptions of how the parents evaluated the child’s responses to and behaviors in relation to the living arrangement in order to enable changes and, thus, optimize their adjustment.

**Table 3:** The resulting themes and categories.

Themes	Categories
***Always free*, *never free***	*Common parenthood without a love relation* *My parenting*, *my way* *Feeling powerless*
**“*Is it right*, *Is it good*?*”***	*Monitoring the child/ren’s reactions* *Adjustments to optimize the practice* *Promoting the child-mother attachment*?

### Always free, never free

Many of the parents described their strivings to have a common parenthood without a romantic relationship. The parents described how they worked to support each other and share both the positive and negative aspects of parenting. Parallel to the striving to communicate and the cooperation efforts, many also spoke about the relief and ease in developing their own parenthood without having to compromise. The coparenting relationship was viewed not only as a present state but as something necessary to keep and grow for the future. Many considered it a natural intention to live close to the other parent during the child’s entire childhood. Communication with the other parent also lessened the parent’s feelings of being cut-off from half the child’s life and those who were left without updates felt powerless and wished for more openness and contact. For some parents, cooperation was not an option. When JPC was due to a court order or when conflicts were intense the parents instead tried to minimize the contact and were sometimes worried about how the children fared in the home of the other parent.

#### Common parenthood without a love relationship

Due to the young age of the children all parents were rather recently separated and for most of them it was an ongoing task to create good working relations.

*“even if the adult relationship doesn’t work*, *parenting may work. Me and X reflect a lot about our parenting because we want our children to fare well and feel secure”*

Mother to a 20 months old girl and a 3.5 years old boy

*” we try to find a balance (in our relationship) where the children are our bond*.*”*

Mother to 18 months and 3 year old girls

*“our efforts (to collaborate) have paid off since the child appears very (emotionally) secure*.*”*

Mother to 3.5 year old boy

Those who split up already during pregnancy or in early infancy were faced with a situation where parenthood basically was their only relation. Ways of creating a working coparenting relationship here were, for example, attending antenatal classes together and allowing the father own time with the infant from very early on to build a relationship.

*”we separated in week 17 of pregnancy. He came to us, in the beginning, 3 evenings a week to establish a kind of attachment relation and I stayed in the background. Since [the baby] was so small I went out for half an hour with the phone if there would be anything acute. Then it happened that I went off for an hour*.*”*

Mother to 3 year old boy

Even in the presence of a new partner the other parent was sometimes regarded as the only person who could fully share some things about the child.

*”only X understands the joy of the little things with [the child], in fact there is no one but him who, like, understands exactly how thrilled and happy one becomes over the little things they say and do*.*”*

Mother to 3 year old boy

Many of the parents described a good relationship with the other parent was as a prerequisite for the child’s adaptation and wellbeing. Mutual confidence was assumed to help children through the separation and bridge the fact that children only spent time with one parent at a time and moved between two different homes.

*”we also communicate through a book in the diaper bag*.*”*

Father to 2 and 4 year old girls

*”we have this journal that we work with. To build trust in our daughter. We always write three things we have done with her so you can mirror that later. Then you have immediately built a bridge of confidence where the children can feel that mum and dad work together”*.

Father to 2 year old girl

Common principles for upbringing or ways to tackle emotional reactions or difficulties in the child’s behavior was sometimes found important.

*”she has been eating poorly in X’s home so we have spoken a bit about food*.*”*

Father to 3 year old girl

The ambitions of communicating ranged from common weekly dinners to strict email or text contact and not meeting in person to avoid open conflict. For the vast majority, however, the contact was intense, with several updates weekly. On the practical side this included shared bank accounts, common birthday parties or coordinated purchases for the child.

*”X buys the winter clothes and I buy the shoes*.*”*

Mother to 3 year old boy

The communication between the parents was also seen as a way of lowering the sense of disconnection from the child.

*”it doesn’t feel like 50% of the children’s lives is like cut off as it would be if we didn’t speak, but that you are decently involved in how they are doing when they are with the other*.*”*

Mother to 2.5 year old boy and older siblings

The parental relationship was viewed not only as a present state but as something necessary to keep and grow for the future. Many considered it a natural intention to live close to the other parent during the child’s entire childhood. Living close to the other parent was regarded as even more important when the children grew older and friends and leisure activities would be more important.

*”one cannot make a lifelong commitment to always be neighbors. But we kind of have the ambition to at least live nearby. Especially when he’s older*.*”*

Mother to 2 year old boy

#### My parenting, my way

Despite the efforts to cooperate many of the parents spoke about the relief, lightness and ease in developing their own parenthood without having to compromise. This was found to facilitate the parent-child relationship and several rated both their own and their ex-partner’s relationship with the child as improved after the separation. Instead of the efforts to compromise in an unsatisfying parental relationship, they could do what suited themselves and their children.

*”even if you’re not stable together you may be that on your own”*.

Mother to 2 and 5 year old boys

But there were also difficulties in being a single parent, at least in times of defiance and development.

”suddenly you’re alone with the disciplining”

Mother to 2.5 year old boy

Many of the parents appreciated that the time without the child contributed to the satisfaction in parenthood.

*”I become a better mother when I get some breathing space and have more energy*.*”*

Mother to 2 year old girl and 4 year old boy

*”I am only a halftime parent, so it doesn’t require the same sacrifices. You get a natural relief in joint physical custody. It seems burdensome to be a single parent*.*”*

Father to 2 year old girl

Before the separation the mother was often perceived as involved also in the father’s relation to the child/ren whereas now it was something entirely between the father and the child. Improved father-child relations as a consequence of the shared parenting arrangement, were frequently described by both mothers and fathers. Some fathers however, felt that their parenting capacity was doubted by the mothers. When fathers felt they now got along well with their child/ren on their own, a sense of relief or, in some cases, revenge, evolved.

*”even if I don’t find JPC optimal for young children I see that X has gotten a much better relation with the children and isn’t just a ‘Sunday Dad’*.*”*

Mother to a 20 months old girl and a 3.5 years old boy

*”our relation (father-child) has rather deepened. As a parent, one is now forced to be constantly present, which isn’t the case when you are two”*.

Father to a 2.5 year old boy

#### Feeling powerless

Some of the parents described feelings of helplessness related to the living arrangement. These feelings could evolve when sharing parenthood happened with someone perceived as impossible to communicate or collaborate with. This feeling could also emerge from the fact that half of the child’s life was out of hand and every second week lonely. For some parents the lack of insight in the children’s lives when they lived with the other parent contributed to the sense of powerlessness. Some children lived with new partners and siblings, whom the other parent did not know, and the children were too young to express themselves about these relations.

*”the longing [for the child] the weeks she doesn’t live here … I try to work the entire Mondays when she is moving to her mother, so I don’t have to think about it*.*”*

Father to a 3,5 year old girl

When the other parent had mental health issues, such as recurrent depression, alcohol or substance abuse some parents monitored their children for signs of neglect. Letting children stay with the other parent on holidays characterized by alcohol and festivities, such as Christmas or Swedish Midsummer, could for example be associated with worry. Others dealt with the situation as a fact they could not change, even if it might be harmful for their children.

*“I got very worried when I got this letter that seemed so depressive. But I took it to the counsellor where we discussed how X could find more support for himself. He has now moved to his parents’, so when he doesn’t have the strength, his parents help out [with the children]*.*”*

Mother of 18 months and 3.5 year old girls

*“they will stay with me for Midsummer’s eve, thanks heaven! Our son has said that mummy smells of liquor so it’s a relief to have them then so I don’t need to worry*.*”*

Father of 2 year old girl and 6 year old boy

Some fathers said they felt a constant threat that the mother would no longer accept the equal arrangement but claim to have the children more. A few mothers expressed being trapped in a situation they could not change and strove to accept.

*”we are each other’s complete opposites regarding everything from vaccination to….everything. And it is in fact a very difficult puzzle. But I think we should live close to each other and find a dialogue, we have to. You cannot rule each other out. (…) for me and (the child) it would be great if the father didn’t exist at all. But now he does and then I know that, yes, we just have to find a solution to this*.*”*

Mother to a 2 year old boy

### Is it right, is it good?

The theme Is it right, is it good? includes descriptions of how the parents evaluated the child’s responses to and behaviors within the living arrangement to enable changes to optimize their adjustment. Such changes included change of schedules or meeting extra with the child/ren if they were missing one parent. Most children’s reactions were judged as normal and as signs of the children’s’ adaptation. Despite this, parents could still worry how the constant moving would affect their children in the long run. Some mothers worried that the children´s attachment relations to them would be negatively influenced while some fathers meant that their ex-partners used this as an excuse to get extra time with the child. In some instances the importance of the mother-child bond was stressed because the father had spent less time than the mother with the child before the separation, whereas other simply held more conservative views about the respective importance of the mother and father to the child in the early years.

#### Monitoring the child’s reactions

Both mothers and fathers expressed a low-grade worry for the child’s wellbeing and described that they were continuously evaluating the child’s behaviors and reactions. It was often perceived as difficult to judge if the child/ren’s behaviors were just developmental phases or signs of reactions to the JPC practice. Often children’s reactions were initially interpreted as reactions to the changes in the family.

*“and what lies beneath his behavior? Is it a reaction to the new situation that his father isn’t there? What does it signify? And how to handle it? I find that difficult. And there maybe I would have reacted differently if I had lived with his father*.*”*

Mother to 2.5 year old boy

*“one asks oneself a lot ´what is right´. Both X and I have read lots of related literature on the internet*.*”*

Father to a 20 months old girl and a 4 year old boy

Despite that many parents meant that shared parenting was the best solution for child/ren, they described an everyday life where signs of the contrary were looked for in their children. Observed child reactions included some children not acknowledging a parent if they called or met during the other parent’s week. Similarly, some children did not share experiences they had with the other parent. Overall, however, the majority of the parents were quite convinced that the child showed no signs of ill-health and had adapted well to the practice.

#### Adjustments to optimize the practice

With cues from the child/ren’s behavior the parents tried to establish or change routines and make adjustments to the practice to improve the child/ren’s situation. Alterations to the schedule to shorter or longer periods with each parent, could be made depending on how the child adjusted.

*“we noticed that [the child] got worried by all the moving back and forth, so then we started with almost whole weeks*.*”*

Mother to a 3 year old boy

Some parents arranged contact boosters if the child/ren were missing one of the parents or if the parents themselves felt that it was too long not to meet for a whole week.

*“if I experience a great longing for the children in the middle of the week when they are at their mother’s or if one notices that the children miss their father so much that they are crying all the time, then you get over there for an hour or two and perhaps go out to play in the park or something*.*”*

Father to a 2 year old girl and school-aged siblings

Parents also described other adjustments to smoothen the children’s’ alternate living. For instance, instead of picking up the child/ren in each other’s homes it could be easier to shift through preschool. Other parents however made a clear statement that the child/ren should see them together and that they should have a coffee or dinner together when shifting homes. Most of the parents described that they tried to keep at least the basic routines for sleep and food similar in both homes in order to facilitate life for the children.

*“C is very tricky with eating and sleeping so the routines have been there since before we separated. We talk and make sure that everything is very similar, from which songs we sing to when to sleep or routines for food*.*”*

Mother to 2.5 year old girl

Due to the intense development in these young children, communication about adjustments in routines and handling of children’s temper and defiance was sometimes regarded as necessary.

*”now the 3 year old is in a period of defiance and there it is very important to have the same values. For the child rearing aspect specifically, we have to have common values*.*”*

Father to 3 year old girl

In some cases, parents described that the children knew that there were different routines in each home and that this might be difficult to adjust to for the child. Some parents had, however, scarce knowledge about the routines in the other home. For some parents this was a source of frustration and worry, while other did not see this as problematic.

*”no, in fact I don’t think so, [that we have similar routines]. Because I think we’re have quite different. (…). I think children are adaptable and understand that mum does it this way and dad that way. You don’t do it the same way even if you live together*.*”*

Mother to a 3.5 year old boy

#### Promoting the child-mother attachment

Some of the mothers thought that the child suffered more from the separations from her than from their father. Others felt guilty for not being with their child all the time, or guilt-tripped by others. Some of the fathers shared the mother’s worries regarding the child’s separation from her, or agreed to what was understood as a general rule. However, the narratives revealed that parents could have mixed intentions. As one father said:

*“we have said that, since [the children] are so young, they shall stay in their mother’s home. I moved to an apartment of my own (…). Practically speaking we have them half of the time, which means they sleep in my place too. But we tell them that they only live in one place, even if they also stay with me*.*”*

Father to a 20 months old girl and a 4 year old boy

Some fathers felt that the mothers used the child’s attachment to her as an excuse to get more time with the child because of the child’s young age. However, this was not always found legitimate.

*”it is obvious that when X says it is not good for [the child] to be away from her more than 3 nights in a row*, *it is obvious that it is SHE who has problems with being away from him”*

Father to a 18 months old boy

Among the very young infants, mothers had trouble being away for too long.

”when we moved apart he was only 7 months old and I felt it was too early and I felt that he couldn’t handle being away from me that much”

Mother to a 3 year old boy

In this example, the couple found a way to gradually increase the time spent in the father’s care letting the mother and the infant ease into the situation during the first six months of separation.

## Discussion

In this interview study we explored parents’ perceptions on how they experience and practice equally shared JPC for their 1–4 year olds. Due to the young age of the children all parents were rather recently separated, with a mean of 21 months since the separation. The sample consisted of a mixture of parents who had either mutually agreed on JPC, decided after mediation or had a court decision prescribing JPC. Two themes emerged in the analysis, one theme describing parenthood after divorce or separation, *Always free*, *never free*, and one theme describing how the parents viewed the child in JPC, *Is it right*, *is it good*?.

A perspective of common parenthood as being separate from a romantic relation emerged in the theme *Always free*, *never free*. The end of the romantic relationship with the other parent was not regarded as a reason for not continuing to coparent. Instead, the underlying assumption that children need both parents on a daily basis, led many parents to strive to bridge the gap between the children’s two homes. The goal was that the children would be emotionally secure and their lives coordinated. Parenting on halftime basis was also found beneficial for the parents themselves. Ease and a sense of freedom, was described, both by being able to parent on their own but also due to the time without the children. The frequent communication with their coparent helped them keep a sense of being present in their children’s lives and not cut-off half of the time. In the second theme, *Is it right*, *is it good*? parents described monitoring the child’s responses to the living arrangement and making changes according to optimize their adjustment. Most parents were pleased with the arrangements with the notable exception of couples experiencing ongoing conflict or having an ex-partner who neglected the children. Even when being generally satisfied with the JPC arrangement, the majority of the informants worried about how the children would be affected by the separation as well as by the frequent moves imposed by JPC. They monitored their children’s behaviors and emotions both in order to adjust the parenting plans to what they perceived were the children’s needs but also to find signs of general reactions to the divorce or the living arrangement. Changes of schedules were made both to avoid frequent separations (i.e making the periods longer) as well as to avoid longing (i.e. making the periods shorter).

### Coparenting after separation

Also for very young children in Sweden JPC has become more common than sole custody, at least among parents with high educational levels [1]. Despite the separation, the coparenting and communication with the child’s other parent was fundamental for parents in this study. From their perspective it was the only possible way of fulfilling the core task of parenting: maintaining the child’s wellbeing and sense of security through ensuring it’s right to close attachment relations to both parents. Some parents also wanted their coparenting relationship to be visible for the child and arranged for dinners or get togethers for the child to perceive the parents’ contact. The expressed belief that coparenting is important for children’s health and wellbeing has, in earlier studies, been shown to boost coparental cooperation and communication [55,56]. Such communication, in turn, further strengthens the parent’s relation [29]. Coleman et al (2014) found that *how* parents spoke provided insight into how they perceived their coparenting relationships. Those who spoke about “we” in parenting tended to be more focused on their children, while those more preoccupied with themselves or their ex-partners more often referred to what ‘‘*I”* did [29]. Similar to this, some parents in this sample referred to their ex-partner as the most important person to discuss their children with, even if they had a new partner.

Regardless of the child’s living arrangement, legal shared custody demands common decisions of the parents since it presupposes that parents agree on all issues of importance for the child [57]. Parent’s ability to coparent and to be engaged, emotionally available and sensitive to their children has been shown to be more important for young children’s wellbeing than number of overnights in the parents’ respective homes [24,42,43]. For the parents in the present study, a working coparenting relationship was assumed to be the bridge between the two homes, helping the child to a greater sense of coherence. Similarly, divorced parents in an interview study by Coleman et al (2014) acknowledged that they consciously chose to put their children’s needs first through regulating their emotions and adjusting communication strategies while negotiating with their coparent, instead of focusing on what they disliked about him or her [29]. Coparenting was also important for practical reasons. Decisions on feeding practices, bed times or tackling of emotional and behavioral issues were often regarded as necessary to agree on because of the children’s young age. Besides the practical caretaking, some parents also tried to match their childrearing such as responses to temper tantrums, defiance and children’s worries. In their classic work Maccoby et al. (1993) showed that common views on discipline and perception of parenting roles were factors that increased the quality of coparenting [58]. When parenting was perceived as difficult and children entered new developmental stages, parents who could communicate discussed what the child needed in terms of responses or routines. Other parents instead chose to accept that they were different from each other in terms of values as well as routines and expected their children to adjust to these differences. Parents diverged in their perceptions whether these adjustments came naturally or were more difficult for the children.

When the coparent relationship did not work, parents expressed feelings of helplessness. This was common when parents had difficulties communicating and collaborating. Parents with low confidence in the other parent’s parenting abilities also described the anguish associated with leaving their children’s in this parent’s care. Confidence in the other parent’s reliability in caring for the children has indeed been identified as an important factor for high quality coparenting [58,59]. Research literature has clearly shown that ongoing parental conflict is detrimental to children’s health and wellbeing [60]. Whether JPC can be beneficial for children when parents lack mutual confidence or have intense conflicts has been debated [36,61]. Recent studies [62] as well as earlier work [63] has suggested that parent-child relationships and children’s health may benefit from JPC even in high conflict situations, while others have pointed out the increased risks for children to be exposed to the conflict in JPC [61]. Coparenting has been shown to be closer linked to child outcomes than other aspects of parents’ relationships and could be one important factor to target with interventions to produce mutual trust or to help parents come to agreement on daily routines and schedules in JPC [64].

### Attachment and JPC

The intensive discussion among experts about children’s wellbeing in JPC is today theory-driven due to the lack of longitudinal studies. With reference to attachment theory [35] some mothers in this study were concerned about the potential stress for the child caused by separation from the mother. Sometimes these mothers arranged extra time with their child to promote the child’s attachment relation. Some of the fathers shared these mother’s worries but nevertheless, fathers and most mothers emphasized the importance of both parents and we have previously reported that most mothers and fathers in this sample regarded JPC as “a given” [53]. In the other aspects described in this manuscript there were however no clear gender differences. It seems plausible that a father’s early engagement in parenting plays an important role in how children fare in JPC. Separations from the mother may involve emotional stress if children are not used to being taken care of by their fathers, whereas a history of close everyday contact can create strong bonds between fathers and their children. This assumption seems to have guided some of the parents when they described how the father was gradually introduced to taking care of the baby. This presumption is also supported by the research literature showing that living together with a parent is strongly and positively associated with the quality of the relationship with that parent [65–67]. In this study some parents described that the father-child relationship had improved after the introduction of JPC. Nevertheless, the literature on JPC and attachment is by no means conclusive and further studies are needed to untangle what type of JPC under what circumstances and within which contexts could benefit or harm children’s wellbeing.

### Methodological considerations

In qualitative research, credibility, dependability, and transferability are prerequisites for scientific quality. Credibility refers to confidence in the truth of the data in relation to the research question; dependability to the stability of the data (reliability of the analytical procedure) and; transferability to applicability of the findings in other contexts.

#### Credibility

The interview guide, the study design, recruitment, and the planned analyses were discussed in multiple research team meetings. The interview guide shaped the interviews so that they all covered the same topics, but probing questions were also employed to explore individual stories.

#### Dependability

Dependability was strengthened by the use of co-analysts, researcher reflexivity, and clear decision trails. All interviews were read, re-read and discussed by three of the authors. Themes were identified by two of the authors during the analyses (co-analyses) and all authors discussed and agreed on the final themes. Possible preconceptions held by each author were discussed before the analyses to minimize the effect of subjective bias (researcher reflexivity). Three of the four authors grew up in Sweden where gender equity in parenthood was considered a strength, from both the child's and the parent's perspectives. These authors also have personal experiences of shared parenting after separation or divorce in different ways (as a child, parent or grandparent). The third author was raised in Hungary, where a more traditional perspective on gender- and parental roles was predominant and with no personal experience of parental separation. The authors discussed their preconceptions in relation to the findings during the entire process of analysis and when preparing the manuscript.

#### Transferability

The parents volunteered to participate by responding to a newspaper ad and the sample is not intended to represent the whole population of Sweden’s non co-habiting parents. Although self-selection introduces certain forms of bias, such as e.g. having strongly positive or negative experiences, the sample includes parents who, either through their own will, mediation or a court order, actually shared custody equally and were willing to disclose about their thoughts and feelings about this to researchers. The parents were also aware that the interviews were conducted by child psychologists and to some the possibility to talk about their children with such an expert was tempting even if it was for research purposes.

Despite its possible selection bias, the sample size of 48 parents is unusually large for a qualitative study. Some of the parents were very positive to JPC, while others were more critical. Compared to the general population the participating parents more often belonged to a high income category and had a college or university degree and the latter was especially true for the mothers. Earlier studies have shown that parents in JPC families more often are highly educated compared to families with single care arrangements [1,3,5,9,17,43]. A wide variety of arrangements and experiences of JPC were represented. To increase transferability, negative case analysis was also employed, meaning that we paid special attention to accounts that differed from the vast body of descriptions. In Sweden, JPC for toddlers has become more common than other post-separation solutions [1]. Being a parent in Sweden might therefore be different than in other parts of the world, despite the universal themes of parenting and parental worries. It is also possible that non-equal shared custody with different proportions of time spent with each parent might have other parental perceptions attached to it. Future studies with representative samples are needed to confirm whether the results represent the general population of parents with JPC.

## Supporting information

S1 File Interview guide. (DOCX)

S2 File De-identified interview excerpts. (DOCX)
