# Supplementary material for: “We also communicate through a book in the diaper bag”—Separated parents´ ways to coparent and promote adaptation of their 1-4 year olds in equal joint physical custody
Source: PLoS One. 2019 Apr 10;14(4):e0214913. doi: 10.1371/journal.pone.0214913 (PMC6457541; doi:10.1371/journal.pone.0214913)
Supplement: S1 File — (DOCX) [file pone.0214913.s001.docx]

**S1 File. Interview guide**

*Questions marked with an X are relevant for the submitted paper*

Code:

**Oral information to participants in interview study on joint physical custody for children under 4 years of age.**

We are researchers on Karolinska Institute and the aim of this study is to reach knowledge on JPC in families with young children that can be useful for individual parents, experts working with separating parents and policy makers.

To particpate you should be parent to at least one child who today is under 4 years of age who lives in JPC. With JPC we mean that a child lives rougly equally much with both parents. If you have more than one child under 4 years you can answer the questions for all those children.

The interview we are conducting explores your views on JPC, your experiences of your child’s wellbeing in JPC and your views on your parenting in general. We will ask concrete questions about your situation. The interview will be tape recorded.

Your participation in the study is voluntary and you can at all times withdraw. If you choose to participate you should know that everything you say will be handed confidentially. It means that no one outside the research group can connect your name with your answers. If you have questions or want further information on the study you are welcome to contact us at all times.

Given this information-do you still want to participate in the study?

I will now start the recording and ask again: Given this information-do you want to participate in this study? (*the consent was recorded*)

___________________________________________________________________________

What is the name of your child? (who lives in JPC and is under 4 years of age)

**Child 1** name ________________ G □ B □ Age: _____________(months)

Do you have other children?

*If you have more than one child who is 4 years or younger and have JPC I will ask questions about each of them. If you have older children I ask you to focus on your younger child when you answer.*

Name:

Address:

Phone:

M F Age:__________

□ □

Education:

Primary school □

Secondary school □

College/University □

Income per month:

Residency:___________________________

**Practicalities:**

How old was the child when you separated? ___________ (When was that?_____________)

For how long have you had JPC?

Have you had another living arrangement since you separated?

How often does the child move?

How do you arrange the moves practically?

X. How do you think the schedule works?

What variances have you tried?

**Attitudes to JPC and parenting**

How come you have JPC? (who wanted it?)

What are your views on gender equality in parenting?/ How do you view mums’ and dads’ responsabilities in parenting?

Do your views on parenting affect the fact that you have JPC? ___________________________________________________________________________

**How does it work?**

X. How do you think JPC works? (compared to living together/single custody?)

What do you think is the benefit of JPC? For you/for the child?

What are the drawbacks of JPC? For you/for the child?

___________________________________________________________________________

**How does your child like it?**

X. How does your child react when you change between the homes?

How do you think it is for him/her to have JPC?

Do you think JPC suits your child? (why/why not?)

Is your child favorizing one of you over the other parent? If so, how do you think your child reacts to being apart from the favorite?

X. Do you think it is important that it is similar in both homes? /that both parents have similar/same routines with the child?

How do you think the time in the other parent’s home is for your child?

X. Do you trust your ex as a parent?

X. Is it important that the parents communicate about the child and parenting?

X. What questions do you think it is important to communicate about? (why/does it work?)

___________________________________________________________________________

**Young children and JPC more in general**

What prerequisites do you think are necessary to make JPC work for young children?

What do you think is important to make a young child feel safe and secure?

X. If you think about young children’s emotional development (for example attachment), how do you think that is affected by JPC?

Do you think there are children that JPC is unsuitable for?

___________________________________________________________________________

**Facts about you**

Do you have a partner?

How much do you earn per month? (taxes excluded)

___________________________________________________________________________**Home**

Is it important to live close to the other parent? Difficult to live near each other?

Did you and the mother/father discuss adjusting your homes to suit JPC?

Does it turn out as planned?

**The child’s other parent:**

M F Age:__________

□ □

Education:

Primay school □

Secondary school □

College/University □

Income per month:

Residency:___________________________

New partner? Yes □ No □

Live together? Yes □ No □

Have children? Yes □ No □

**Children under 4 in JPC:**

**Child 2**

name ________________

Girl □ Boy □

Age: _____________(months)

**Child 3 Child 4**

name ________________ name ________________

Girl □ Boy □ Girl □ Boy □

Age: _____________(months) Age: _____________(months)

**Siblings over 4 or who don’t have JPC:**

**Sibling 1 Sibling 2**

name ________________ name ________________

Girl □ Boy □ Girl □ Boy □

Age: _____________(months) Age: _____________(months)

Live with the family Live with the family

Yes □ no □ sometimes □ Yes □ no □ sometimes □

Full sibling □ Full sibling □

Half sibling □ Half sibling □

Not biological siblings □ Not biological siblings □
