# Supplementary material for: “We also communicate through a book in the diaper bag”—Separated parents´ ways to coparent and promote adaptation of their 1-4 year olds in equal joint physical custody
Source: PLoS One. 2019 Apr 10;14(4):e0214913. doi: 10.1371/journal.pone.0214913 (PMC6457541; doi:10.1371/journal.pone.0214913)
Supplement: S2 File — (DOCX) [file pone.0214913.s002.docx]

# S2 File. De-identified interview excerpts

**Common parenthood without a love relationship**

*“even if the adult relationship doesn’t work, parenting may work. Me and X reflect a lot about our parenting because we want our children to fare well and feel secure”*

Mother to a 20 months old girl and a 3.5 years old boy quoted from: I have seen other who think they have exclusive right to the children when they get divorced, but you need to separate your own conflicts with the other parent from the children’s relationships with that parent. Even if the adult relationship doesn’t work, it does not mean that parenting does not work. Me and X are thinking pretty much about our parenting because we want the children to fare well and feel secure. (Jag har sett andra som tagit för givet att dom har någon ensamrätt till barnen när dom separerar, man måste kunna hålla isär sin egen konflikt till den ena föräldern ifrån barnens relation till föräldern. Den vuxna relationen kanske inte fungerar men det behöver inte betyda att föräldraskapet inte fungerar. Jag och X funderar ganska mycket kring vårt föräldraskap för vi vill att barnen ska må bra och känna sig trygga (A27).)

*” we try to find a balance (in our relationship) where the children are our bond.”*

Mother to 18 months and 3 year old girls quoted from: Then I have also had the idea that we should do something together all of us, which we have done and it works well. I do not think it is sensitive to socialize a little sometimes. We try to find a balance where the children are our bond and that it is obvious that we celebrate together. (Sen har jag också haft som idé att vi skulle göra något tillsammans allihop, vilket vi har gjort och det fungerar bra. Jag tycker inte att det är känsligt att umgås lite ibland. Vi försöker hitta en balans där barnen är vårt band och att det är självklart att vi firar tillsammans (A20).)

*“our efforts (to collaborate) have paid off since the child appears very (emotionally) secure.”*

Mother to 3.5 year old boy quoted from: We communicate well, we help each other and strive to still share, not only the practical, but actually share the positive parts (of parenting)…. The efforts have paid off since C appears very secure. There have been no strong reactions and they, I do not see them as sad or worried, but they handle it great, great. Much better than I do myself. Yes, it has been good but at the same time, on a personal level, it has been difficult because now it has been a very long time, it has been very many months.… I have the same type of relationship (to X) that we had before, only that we don’t sleep in the same place. (Vi kommunicerar bra, vi hjälper varandra och stävar efter att vi fortfarande ska dela, inte bara det praktiska, faktisk dela dom positiva bitarna. Dom ansträngningarna har betalat sig i form av att barnen faktiskt känns väldigt trygga, det har inte blivit några kraftiga reaktioner och dom, jag upplever dom inte som ledsna och oroliga utan dom hanterar det jätte, jättebra, mycket bättre än vad jag själv gör. Ja, det har varit bra men samtidigt på ett personligt plan har det varit jobbigt för nu har det gått väldigt lång tid, det har gått väldigt många månader. Jag har ju haft samma typ av relation som vi hade innan bara att vi inte sover på samma ställe (A12).)

How we fare as adults doesn’t matter. The children shouldn’t suffer because things didn’t work out between us. (Hur vi själva mår är skitsamma. Barnen ska inte behöva lida för någonting som inte fungerat mellan oss (B1).)

It is that ambition we have-that as much as possible should be as it was for the kids. We have had that as a common thread all the time-that they should not feel that it is different. And I think we’ve been successful in doing that. (Det är den ambitionen vi har- att så mycket som möjligt ska vara likt för barnen. Det har vi haft som en rödtråd hela tiden -att dom inte ska känna att det är annorlunda. Och det tror jag vi har lyckats med (A23).)

*”we separated in week 17 of pregnancy. He came to us, in the beginning, 3 evenings a week to establish a kind of attachment relation and I stayed in the background. Since [the baby] was so small I went out for half an hour with the phone if there would be anything acute. Then it happened that I went off for an hour.”* Mother to 3 year old boy quoted from: In week 17 I learned that X had betrayed me. I never wanted to see him again but I realized that C had the right to his dad. I had to nag on him to make him come to the parent information and to listen to the baby’s heart sound. Apart from that I never saw him during pregnancy. He was tied down by his new girlfriend. She didn’t want us to meet. I nagged on him to come when I was giving birth so he could tell the story for his son about how he was born if something would happen to me. He was there, I would have made it without him but I think it was nice for our son to see that his father was there. Then he came to us, three evenings a week to establish an attachment to C, and I stayed in the background. It was always in my place so C should feel safe in the home environment. C was so little that I went away for half an hour with the phone if something acute would happen. Then it happened that I went away for an hour. When C was four months he started to eat solids so they could go out. X picked him up and they went out for an hour or two in the baby carrier. It worked well. When C was six months I started to leave C with X for like two hours and he could bring him home to his place. Finally it was a whole day and a whole night when I stopped breastfeeding at seven months. Then we built it up gradually and X had him for two days in a row. C started preschool/kindergarten when he was 1,5 years and then he still stayed with X for two days, we kept that going. Then we started with him being with X almost every second week. We also tried a version where C stayed with me the Wednesday night and vice versa so it wouldn’t be such long periods. It worked until we noticed that C became vary from all the back and forth so we started with almost whole weeks. (När jag var i vecka 17 fick jag reda på att X hade bedragit mig. Jag ville egentligen aldrig mer se X men insåg att B hade rätt till sin pappa. Jag som fick tjata för att få honom att komma på föräldrainformation och att lyssna på barnets hjärtljud. Annars sågs vi inte så mycket under graviditeten. Han var bakbunden av sin nya tjej. Hon ville inte att vi skulle ses. Jag tjatade på honom att han skulle vara med på förlossningen för att kunna berätta historien för sin son hur han kom till världen ifall det något skulle hända mig. Sen var han med, jag hade klarat mig utan honom men jag tror att det är fint för vår son att kunna se att pappa var där. Sen kom han hem till oss i början, tre kvällar i veckan för att skapa en anknytning till B, och då höll jag mig i bakgrunden. Det var alltid hemma hos mig så att B skulle känna sig trygg i hemmiljön. B var så liten så jag gick därifrån en halvtimme med telefonen på ifall det skulle vara något akut. Sen blev det att jag gick iväg en timme. När B var fyra månader åt han smakportioner så då kunde de gå ut och gå då kom X kom och hämtade honom och de gick ut en timme eller två i sele, det fungerade bra. När B var sex månader så började jag lämna honom ett par timmar till X och han fick ta med B hem till sig. Tillslut blev det en hel dag och sen en hel natt när jag slutade amma vid sju månader. Sen byggde vi på gradvis och X hade honom två dagar i sträck. B började på dagis när han var runt 1,5 år och då var han fortfarande hos X två dagar, vi fortsatte med det. Då började vi köra att han var hos X nästan varannan vecka. Vi provade även på en variant där B var hos mig på onsdagsnatten och tvärtom, så att det inte skulle bli så långa avbrott. Det fungerade fram tills att vi märkte att B började bli orolig av allt hattande, så då började vi med nästan hela veckor (A21).)

*”only X understands the joy of the little things with [the child], in fact there is no one but him who, like, understands exactly how thrilled and happy one becomes over the little things they say and do.”* Mother to 3 year old boy quoted from It feels good that it has been a gradual transition in many ways because it is such a terribly big change and for me it is really tough. So in a way I think, I especially think, that it has been good for C that it has been so gradual. For our child is super cool in this. (He) takes it actually so calmly that I sometimes feel: God, shouldn’t he react more? And then I try to think and see that we have to congratulate ourselves and realize that we have done a very good job. Although we have had a lot of trouble and made the most difficult decision you can ever make, we have succeeded in not burdening our child with it…. only X understands the joy of the little things with [the child], in fact there is no one but him who, like, understands exactly how thrilled and happy one becomes over the little things they say and do. (Jag tycker att det är skönt att det har varit en gradvis övergång på många sätt eftersom det är en sådan fruktansvärt stor omställning och för mig är den jättetuff. Så på sätt kan jag tycka, framförallt kan jag tycka att det är, och har varit bra för B att det har varit så gradvis. För vårt barn är supercool i det här. Tar det liksom så lugnt att jag ibland bara men gud ska han inte reagera mer. Och då försöker jag tänka och se att där får vi klappa oss axeln och se att vi har gjort ett väldigt bra jobb. Fast vi har haft det skitjobbigt och fattat det svåraste beslutet man någonsin kan behöva fatta så har vi lyckas med att inte belasta barnet med det. Alltså det finns ju ingen annan än han som kan förstå precis hur förtjust och lycklig man kan bli över små saker dom säger och gör liksom. Allt sådant (A12).)

*”we also communicate through a book in the diaper bag.”* Father to 2 and 4 year old girls quoted from: (Vi kommunicerar även med en bok i skötväskan. Det är sagt att vi ska skriva vad barnen gör i den, inga sakfrågor (B8).) When I said I wanted to leave her, she decided that we should go to a divorce counsellor and we went and talked to a therapist, so then we communicated. But then it was about trying to establish a communication that could work after I moved out. Then, when I moved out, we went to couple therapy to decide on all the daily routines. So the daily routines we have talked about, but there will be problems if you want to deviate from them. It is a 10-page agreement where we have listed all possible situations, but if you want to deviate from them, we can hardly talk to each other. We have decided not to deviate from this agreement during the next six months, until the most painful phase has passed. We also communicate through a book in the diaper bag. It was said that we shall write what the children do in the book, no factual questions, but rather what the children have done so I can talk to them about it without having to ask what they have done at their mother’s during the weekend. (När jag sa att jag ville lämna henne bestämde hon att vi skulle gå på separationsterapi och vi gick och pratade med en terapeut, så då kommunicerade vi. Men då handlade det om att vi skulle försöka få en kommunikation som kunde funka efter det att jag flyttat ut. Sen i samband med att jag flyttat ut sa har vi varit på samarbetsavtal på familjerådgivningen, då har vi benat ut alla dagliga rutiner. Så dom dagliga rutinerna har vi pratat ut om men det blir problem om man skulle vilja avvika. Det är ett 10 sidor lång överenskommelse där vi har spaltat ner alla möjliga situationer, men skulle man vilja avvika från dom så kan vi knappt prata med varandra. Vi har bestämt att inte avvika från dom det närmaste halvåret, tills smärtfasen har gatt over. Vi kommunicerar även med en bok i skötväskan. Det är sagt att vi ska skriva vad barnen gör i den, inga sakfrågor, utan mer vad barnen har gjort så att jag kan föra en dialog med dom om det utan att behöva fråga vad dom har gjort hos sin mamma i helgen (B8).)

*”we have this journal that we work with. To build trust in our daughter. We always write three things we have done with her so you can mirror that later. Then you have immediately built a bridge of confidence where the children can feel that mum and dad work together”.*

Father to 2 year old girl quoted from: It is also important that you are motivating and don’t badmouth the other (parent). That you don’t picture anything that is not true to the children. We have this journal that we work with. To build trust in our daughter. We always write three things we have done with her so you can mirror that later. Then you have immediately built a bridge of confidence where the children can feel that mum and dad work together. And I think that is important, that children who live in JPC feel the confidence. My son’s mother doesn’t want a journal and can’t even talk to me on the phone while C’s mother called the other day and we spoke for 45 minutes. (Det är även viktigt att man är motiverande och inte pratar illa om den andre, att man inte målar upp någonting som inte gäller barnen framför barnen. Vi har en loggbok som vi arbetar mycket med för att skapa ett förtroende hos dottern och i den skriver vi alltid tre saker som man gjort med B så kan man spegla det sedan och prata om det med B. Då har man direkt byggt en bro med förtroende där barnen kan känna att mamma och pappa fungerar ihop och det tycker jag är viktigt, att barnen som bor växelvist känner ett förtroende. Och det tycker jag är viktigt, att barnen som bor växelvist känner ett förtroende. Sonens mamma vill inte ha en loggbok och kan inte ens prata i telefon med mig medan Bs mamma ringde mig häromdagen och vi pratade i 45 minuter. (B16).)

*”she has been eating poorly in X’s home so we have spoken a bit about food.”* Father to 3 year old girl-a quote from: We talk to each other and send text messages. But most often we speak on the phone. (We speak) once a week and when C is there I usually call and X does the same. She has been eating poorly in X’s home so we have spoken a bit about food. I don’t think we should make it into such a big deal. Bedtimes we have discussed, but it is slightly different in her two homes. She goes to bed later at X’s because working hours are different. (Vi pratar med varann och SMS. Men oftast pratar vi med varann i telefon. (Vi pratar) en gång i veckan nar B är där brukar jag höra av mig, X gör samma sak. Hon har ätit ganska dåligt hos X, sa vi har pratat litegrann om mat. Jag tycker inte att vi ska göra en så stor grej av det. Läggtider har vi också haft diskussioner om, men hon har lite olika på båda ställena. Hon lägger sig senare hos X for att det är annorlunda jobbtider (B10).)

We save the child allowance for C. We probably say that when one buys an overall or shoes and so we can use that money. But until today we have saved all that money. (Sparar barnbidraget till B. Vi pratar nog om att när man köper overall och skor och lite så att vi kan ta av dom pengarna eller så. Men fram tills idag har vi sparat alla pengar (A8).)

*”X buys the winter clothes and I buy the shoes.”* Mother to 3 year old boy a quote (X köper overallen och jag köper skorna (A11).)

The shoes we buy together, and the outerwear. (skor det köper vi tillsammans, och ytterkläder (A3).)

*”it doesn’t feel like 50% of the children’s lives is like cut off as it would be if we didn’t speak, but that you are decently involved in how they are doing when they are with the other.”*

Mother to 2.5 year old boy and older siblings a quote from: We have quite a lot of contact. We speak more or less every day. Sometimes it is only that we call to say good night to the kids. Yes, (we talk) almost every day (even when they don’t live here). Call in the evening of I’m not out on something, it doesn’t have to be everyday but basically. And then also sometimes there is something “Oh, we need diapers for preschool and can you bring those trousers?” and then you send a text or an email. And we also usually share if you do something nice out with the kids, you take a nice picture and send to each other. So it doesn’t feel like 50% of the children’s lives is like cut off as it would be if we didn’t speak, but that you are decently involved in how they are doing when they are with the other. We talk about things like when he should stop eating formula during the night and now he starts going more and more to the toilet and then perhaps you should handle it this way or that. You he is afraid of taking a shower or a bath, how do we handle that? With (the older sibling) it is more limit setting since he is a bit more in his pre-teens, from times to times he has quite an attitude, how do we handle it so we do more or less the same things. We talk about that type of things. (Vi har ganska mycket kontakt. Vi hörs i princip varje dag, ibland är det bara att man ringer och säger godnatt till barnen. Ja, (vi hörs) nästan varje dag (även när de inte bor hos en). Ringer på kvällen om det inte är så att man är iväg på något, det måste inte vara varje dag men i princip. Och sedan också att, ibland är bara någonting ”Å, det behövs blöjor till dagis och kan du ta med dom byxorna?” och då skickar man ett sms eller ett mail. Och vi brukar också dela med oss om man är ute och gör något roligt med barnen, så tar man en fin bild så skickar man den till varandra sådär. Så det känns inte som om 50% av barnens liv är helt bortklippt liksom, det kan man tänka sig att det kan vara om man inte pratar med varandra. Utan man är hyfsat delaktig hur det är med dom när dom är hos den andra. Vi pratar om sådana saker som när han skulle sluta äta välling på natten och nu börjar han mer och mer börja gå på toaletten och då kanske man ska göra si och så. Om man är rädd för att duscha och bada, hur ska vi hantera det då. Med (storasyskonet) är det kanske mer gränssättning för han är lite mer i minitonåren, periodvis han har varit ganska så mycket attityd, hur ska vi hantera det då så att vi gör ungefär likadant och så. Det pratar vi om (A9).)

We fulfill the expectations: communicate, help each other, share the positive. We have daily contact. Don’t want the children to have two separate lives. Important not to feel cut off the week you dont have the children. Both parents should know what C have done. One should be updated when C come. (Vi uppfyller förutsättningarna: kommunicerar, hjälps åt, dela det positiva. Vi har daglig kontakt. Vill inte att barnen ska leva två skilda liv. Viktigt inte känna sig bortkopplad den vecka man inte ses. Båda föräldrarna ska ha koll på vad B har gjort. Man ska va uppdaterad när B kommer (A12).)

*”one cannot make a lifelong commitment to always be neighbors. But we kind of have the ambition to at least live nearby. Especially when he’s older.”* Mother to 2 year old boy quoted from: We have said that as long as he is in preschool we will continue to live here. One cannot make a lifelong commitment to always be neighbors. But we kind of have the ambition to at least live nearby. Especially when he’s older to make it easier with his school and also later when he’ll have a group of friends. One can live on the same side of the city at least. I think it is really good for C that we live so close, he recognizes the neighborhood. (Vi har sagt att så länge han går på dagis så kommer vi bo kvar här. Man kan ju inte göra ett livslångt commitment att man ska bo granne med varandra. Vi har väl den ambitionen att man ska bo i närheten i alla fall just att det blir enklare med skolan och även senare när han får kompis umgänge. Man kanske kan bo på samma sida av staden i alla fall. Jag tror att det är jättebra för B att vi bor så nära, han känner igen miljön (B17).)

We don’t live in the same neighborhood but it is about 5-10 minutes on the bike. It facilitates a lot to live close to each other. (Vi bor inte i samma område men det är kanske 5-10 minuter cykelväg. Det underlättar väldigt mycket att bo nära varandra (B18).)

She pressed very early on that I would move closer to them, which I agree that I should but I cannot just move and take any flat, I felt then. Her new boyfriend lives in that area so of course they didn’t want to leave that neighborhood and I agreed with them. (Hon pressade på väldigt tidigt att jag skulle flytta närmare dom, vilket jag håller med om att jag skulle men jag kan ju inte bara flytta och ta vadsomhelst kände jag då. Hennes nya killes bor i det området så då var det klart att dom inte skulle flytta ifrån det området vilket jag höll med om (B18).)

As I said, we live 10 minutes from each other, we have chosen that. We think it is best. It gives C a sense of security. Even if his lives in two homes he lives in one area. (Vi bor som sagt 10 min ifrån varandra, det har vi valt. Vi tycker att det passar bäst. Det ger nog C en trygghet. Även om han bor på två ställen bor han i samma område (A2).)

Today we live 10 minutes walk from each other. It is practical but it ca also feel as ones private life is a bit suffering, as one can bump in to each other. (Idag bor vi ungefär 10 min gång ifrån varandra. Det är praktiskt men kan också kännas som att ens privatliv blir lite drabbat, man kan stöta på varandra (A4).)

**My parenting, my way**

*”even if you’re not stable together you may be that on your own”.* Mother to 2 and 5 year old boys quoted from: The older boy has been wetting his pants but I don’t think that would have lessened if he lived with one parent or only had every second weekend because that is what he has under his belt. I think it is important that there are two stable homes that can give the children what they need. Even if you’re not stable together you may be that on your own*.* I also see that it has worked better for C than for his older brother, because he hardly remembers what happened when we live together as his older brother does. Aldo, C has never had the full attention of both parents as his older brother and thus doesn’t demand it when certain situations arise and there it is more difficult for the older one. (Den stora pojken har haft problem med att kissa på sig och så men jag tror inte att det hade minskat om han bodde hos den ena föräldern eller bara varannan helg för det är vad han har med sig i bagaget. Annars tycker jag att det krävs två stabila boenden som kan ge barnen vad de behöver. Även fast man inte är stabil tillsammans så kan man vara det var för sig. Jag ser även när B var liten att det har funkat bättre för honom än för storebror, för han minns nog knappt vad som hände när vi bodde ihop men det gör storebror. B har heller aldrig haft full uppmärksamhet av båda föräldrar som storebror har och kräver den då inte lika mycket när vissa situationer kommer upp och där är det svårare för storebror (A18).)

*”suddenly you’re alone with the disciplining”* Mother to 2.5 year old boy quoted from:

Suddenly you’re alone with the disciplining. Suddenly you have to make all the decisions yourself. And that is a challenge. (Plötsligt står man väldigt ensam med det här med gränssättandet. Plötsligt så måste man ta alla beslut själv. Och det är ju en prövning (A8).)

*”I become a better mother when I get some breathing space and have more energy.”*

Mother to 2 year old girl and 4 year old boy quoted from: The major advantage for both kids is that their father has been forced to be a full-blown parent, which he wasn’t before we separated. When they are with him he must take the full responsibility himself. I think they have gotten a better relationship with him and that their relation to their father has gained from that. I become a better mother when I get some breathing space and have more energy. (Den största fördelen för båda barnen är att deras pappa tvingats bli förälder fullt ut vilket han inte var innan vi separerade. När de är hos honom så måste han ta ansvar för dem själv. Jag tror de har fått en bättre relation med honom och deras relation till sin pappa har vunnit på det. Jag blir en bättre mamma när jag får andrum och orkar mer (A27).)

*”I am only a halftime parent, so it doesn’t require the same sacrifices. You get a natural relief in joint physical custody. It seems burdensome to be a single parent.”*

Father to 2 year old girl quoted from: The advantages from living together is that you get some time on your own. I am only a halftime parent, so it doesn’t require the same sacrifices. You get a natural relief in joint physical custody. It seems burdensome to be a single parent. (Fördelarna mot att leva tillsammans är att man får lite egen tid. Jag är ju bara förälder på halvtid, så det blir inte samma uppoffringar. Man får naturlig avlastning när man har växelvist, det verkar belastande att vara ensamstående förälder (B3).)

*”even if I don’t find JPC optimal for young children I see that X has gotten a much better relation with the children and isn’t just a ‘Sunday Dad’.”* Mother to a 20 months old girl and a 3.5 years old boy quoted from Even if I don’t find JPC optimal for young children I see that X has gotten a much better relation with the children and isn’t just a ‘Sunday Dad’. (Även fast jag inte tycker att växelvist är optimalt för yngre barn så ser jag ändå att X har fått en mycket bättre relation till barnen och är inte bara en söndagspappa (A27).)

*”our relation (father-child) has rather deepened. As a parent, one is now forced to be constantly present, which isn’t the case when you are two”.* Father to a 2.5 year old boy quoted from: Our relation (father-child) has rather deepened. As a parent, one is now forced to be constantly present, which isn’t the case when you are two. The phase of insecurity, that is what’s hardest. That someone might change her mind or a lurking conflict. To be a father is a bit like…having the system against you. It is elf evident that both parents should be equal. (Vi har snarare fått en mer fördjupad relation. Man tvingas att vara konstant närvarande som förälder. Det behövs ju inte när man är två. (Det svåraste är fasen med osäkerheten, att någon ändrar sig eller att det ligger en konflikt bakom hörnet. Det är det jag upplevt som jobbigast. Och att vara pappa är lite.. man har systemet emot sig. Det är klart att båda föräldrarna ska vara likvärdiga

(B12).)

**Feeling powerless**

*”the longing [for the child] the weeks she doesn’t live here... I try to work the entire Mondays when she is moving to her mother, so I don’t have to think about it.”*

Father to a 3,5 year old girl quoted from The longing the weeks she doesn’t live here is the worst. Emotionally the worst. I try to work the entire Mondays when she is moving to her mother, so I don’t have to think about it. (Saknaden de veckorna jag inte har B är det jobbigaste. Känslomässigt är det de jobbigaste. Jag försöker jobba hela måndagarna när hon flyttar till sin mamma så jag slipper tänka på det (B11).)

I work more Thursday-Friday and less in the beginning of the week when I have C so it has worked well to adjust it. It can feel a bit empty. When you have just left them, that day, it is a kind of emptiness and one is slightly sad and feel the longing for the. But what is hard is the longing for them. (Jag jobbar mer torsdag-fredag och mindre i början av veckan när jag har B så på så sätt så har det gått att anpassa det bra. Det kan kännas lite tomt. När man precis har lämnat över dom, den dagen, det blir någons slags tomhet och så blir man lite ledsen och känner att man längtar efter dom. Men det som är jobbigt är ju att man längtar efter dom (A15).)

It works, but of course one misses one’s daughter when you leave her. The hardest bit is when you leave on the Monday and then shall go to work. The thought that you are not going to pick up your daughter. It is hard to miss your own children, and think about not going to have her the entire week. (Det funkar, men det ar så klart man saknar sin dotter nar man lämnar henne. Den jobbigaste biten är nar man lämnar på måndagen och sen ska gå och jobba, tanken på att man inte ska hämta sin dotter. Det är jobbigt att sakna sitt egna barn, och tänka att man inte ska ha henne hela veckan (B7).)

*“I got very worried when I got this letter that seemed so depressive. But I took it to the counsellor where we discussed how X could find more support for himself. He has now moved to his parents’, so when he doesn’t have the strength, his parents help out [with the children].”* Mother of 18 months and 3.5 year old girls quoted from: We usually have some contact through texts about what has happened during the day. The latest was that X contacted me and described a conflict that has arisen with C at the preschool and we talked a bit about that. And we talk about practical stuff. When C was sick one day I asked X if he could take her to the doctor and he did that without hesitation, and if he has something he needs to do one day I’ll take the children. I think it works well, if there is something we talk about it. I got very worried when I got this letter that seemed so depressive. But I took it to the counsellor where we discussed how X could find more support for himself but also what I can trust works with the kids. There is where my greatest concern has been. He has now moved to his parents’, so when he doesn’t have the strength, his parents help out with the children. (Vi brukar ha lite sms-kontakt om vad som har hänt under dagen. Senast nu så hörde X av sig till mig och berättade om en konflikt som hade uppstått med B på dagis och det pratade vi lite om. Och sen pratar vi om praktiska saker. När B blev sjuk en dag så frågade jag X om han kunde ta henne till vårdcentralen och det gjorde han utan problem, och om han har något han behöver göra så tar jag barnen den dagen. Jag tycker det fungerar bra, är det något så pratar vi om det. Jag blev väldigt orolig där ett tag när jag fick ett brev som var väldigt depressivt men då tog jag med det till familjerådgivaren där vi diskuterade fram hur X kunde få mer egen hjälp och stöd men vad jag också kan lita på fungerar med barnen, det är där min största orolighet har legat. Han har flyttat till sina föräldrar nu så när han inte orkar kan de hjälpa honom (med barnen) (A20).)

*“they will stay with me for Midsummer’s eve, thanks heaven! Our son has said that mummy smells of liquor so it’s a relief to have them then so I don’t need to worry.”* Father of 2 year old girl and 6 year old boy quoted from*:* Last Christmas I had them the entire week of Christmas and she had them the whole week over new year’s eve. They will stay with me for Midsummer’s eve, thanks heaven! Our son has said that mummy smells of liquor so it’s a relief to have them then so I don’t need to worry. She drinks a lot. (Den julen som var så hade jag dom hela julveckan och hon hade hela nyårsveckan. Jag har dom på midsommar tack och lov. Sonen har påtalat att mamma luktar sprit så det känns skönt att ha dom då så man slipper oroa sig. Hon dricker väldigt mycket (B13).)

*”we are each other’s complete opposites regarding everything from vaccination to….everything. And it is in fact a very difficult puzzle. But I think we should live close to each other and find a dialogue, we have to. You cannot rule each other out. (…) for me and (the child) it would be great if the father didn’t exist at all. But now he does and then I know that, yes, we just have to find a solution to this.”* Mother to a 2 year old boy quoted from: I advocate an LCHF diet, so more fat less carbohydrates and also an organic diet, while X runs with light products and no fat. We are each other's opposites regarding everything from vaccinations to… .everything. And it is in fact a very difficult puzzle. But I think we should live close to each other and find a dialogue, we have to. You cannot rule each other out. If I should speak for myself I would say “I think he is an asshole” because he left me during pregnancy and I even had had feelings for him. But apart from what I think of him as a person, for me and C it would be great if the father didn’t exist at all. But now he does and then I know that, yes, we just have to find a solution to this. I think we should live close to each other and we need to find a dialogue, we need it. One cannot exclude each other. And be able to trust each other. If there is a trust, there is a communication, there is a dialogue so that one can help each other to structure life so there will be a way around the child. *(*Jag förespråkar en LCHF- diet alltså mera fett mindre kolhydrater och dessutom ekologisk kost, X kör med light produkter och inget fett. Vi är varandras motsatser i allt från vaccinationer till….allt. Och det är svårt att få ihop. Jag tror att vi borde bo nära varandra och vi måste hitta en dialog, vi måste det. Man kan inte utesluta varandra. Om jag ska prata utifrån mig skulle jag säga ”Jag tycker (pappan) är en skitstövel” som lämnade mig med graviditeten och då hade jag dessutom känslor för honom. Men bortsett vad jag tycker om honom som person skulle det vara mycket lättare om han inte fanns, mycket, mycket lättare. Och kunna lita på varandra. Om det finns ett förtroende, finns en kommunikation, finns en dialog så man kan hjälpa varandra att överbygga så det blir en väg kring barnet (A10).)

If you should have JPC is should be a demanded that parents could communicate and convey things to each other, because he can’t do that. (Om man ska ha växelvisboende så ska det ska vara ett krav att föräldrarna kan kommunicera och förmedla saker till varandra, för han kan ju inte det (A10).)

**Is it right, is it good?**

**Monitoring the child’s reactions**

“*and what lies beneath his behavior? Is it a reaction to the new situation that his father isn’t there? What does it signify? And how to handle it? I find that difficult. And there maybe I would have reacted differently if I had lived with his father*.” Mother to 2.5 year old boy quoted from: What lies beneath his behavior? Is it a reaction to the new situation that his father isn’t there? What does it signify? And how to handle it? I find that difficult. And there maybe I would have reacted differently if I had lived with his father. It is unknown territory, it is the first child which in itself is a challenge. And he has like a rather hot temper and with all these changes that have come, with two homes and suddenly mum is kissing someone else and dad is kissing someone else, of course it is quite a lot for him to take in. At the same time it is also, but maybe I am just protecting myself, a very rewarding age to separate at because it is before they can feel any guilt at all and he has still formed attachment bonds to both me and his dad and he, like, has some sort of security and is like very curious about his surroundings so he easily takes in. People who likes him, he likes back. And the people I like, he likes too. So he is in a very rewarding age I suppose, but there might be a protection in all this too. I mean, one only wants to read positive things about separations. (Vad står hans beteende för, är det en reaktion på den nya situationen att pappa inte är där? Vad handlar det om? Och hur man ska bemöta det, det tycker jag är svårt. Det hade kanske varit annorlunda om jag levt med hans pappa. Det är väldigt okänd mark, dels att det är första barnet det i sig är bara en utmaning. Och han har liksom ett ganska hett temperament och med all den här omställningen som har blivit, med två boende och helt plötsligt pussar mamma någon annan och helt plötsligt pussar pappa någon annan, det är klart att det är mycket för honom. Samtidigt är det också, men det kanske jag bara skyddar mig bakom att det är en väldigt tacksam ålder att separera för att, det är före dom kan känna någon skuld över huvud taget och han har ändå knutit an till både mig och pappan och han har väl någon sorts trygghet och är väldigt liksom nyfiken på sin omvärld så han tar ju lätt in, så folk som gillar honom gillar han tillbaka, och sådana personer jag gillar, gillar ju han. Så det är på så sätt en väldigt tacksam åder, föreställer jag mig, det är kanske också skydd också i allt det här. Jag menar man vill ju bara läsa positiva saker om separation (A8).)

One wonders what would be best from the child’s perspective. (Man undrar vad som kan vara bäst utifrån sitt barns perspektiv (A8).)

Of course I think about whether it is right or not. (Det är klart att man tänker på om det är rätt eller inte (A25).)

I think it is important that you do it right in a way. But of course that is individually. It is difficult stuff and difficult decisions. You do it as good as you can. (Jag tycker att det är viktigt att man gör rätt på något vis. Men det är klart att det är individuellt. Det är svåra grejer och svåra beslut. Man gör ju så gott man kan (A25).)

“*one asks oneself a lot ´what is right´. Both X and I have read lots of related literature on the internet*.” Father to a 20 months old girl and a 4 year old boy quoted from: One asks oneself a lot what is right*?* We read lots of related literature on the internet and took in all advice we could find. *(*Man frågar sig ju vad som är rätt hela tiden. Vi läste runt på internet och tog till oss alla råd som finns (B4).)

We have been on these parenting counselling sessions. (Vi har varit på sådana här föräldrasamtal (A13).)

We used the last sessions on the psychiatric counselling to talk about parenting. (Vi använde de sista samtalen hos psykhälsan till att prata om föräldraskapet (A13).)

I called Save the Children’s helpline. (Jag ringt rädda barnens föräldratelefon (A3).)

**Adjustments to optimize the practice**

“*we noticed that [the child] got worried by all the moving back and forth, so then we started with almost whole weeks*.” Mother to a 3 year old boy quoted from: One week is such a long time in a small child’s world so we decided that C should stay 2-3 nights and then move so he would grasp the concept of changing (homes). But we noticed that C got worried by all the moving back and forth, so then we started with almost whole weeks. We noticed that all the good byes were hard for him. After that he stayed whole weeks and it was much better. He stayed 2-3 nights during two months. (En vecka är lång tid i ett så litet barns värld så vi bestämde oss för att B skulle bo 2-3 nätter och sen flytta då att han skulle fatta grejen med att byta. Men vi märkte att det blev jobbigt för M med så mycket avsked". Efter det bodde han en hel vecka, och det gick mycket bättre. Han bodde 2-3 nätter under två månader (A18).)

When C was little the mum should have C and I see her a few days and then leave her with the mum again, but it was to much back and forth so now we have Monday to Monday. (När B var liten skulle mamman ha B och jag träffa henne några dagar och sen lämna tillbaks henne, men det blev så hattigt så nu kör vi måndag till måndag (B7).)

“*if I experience a great longing for the children in the middle of the week when they are at their mother’s or if one notices that the children miss their father so much that they are crying all the time, then you get over there for an hour or two and perhaps go out to play in the park or something*.” Father to a 2 year old girl and school-aged siblings quoted from:

Try to squeeze in one day a week when I visit them at their mum’s. The opitmal is if I experience a great longing for the children in the middle of the week when they are at their mum’s or if one notices that the children miss their father so much that they are crying all the time, then you get over there for an hour or two and perhaps go out to play in the park or something. But then you have to make it clear to the kids that “now daddy comes for an hour or two because I see that you miss your daddy”. (Försöker klämma in barnen en dag i veckan (hälsar på dem hos mamma). Det optimala är att om jag har väldigt stor saknad till barnen när dom är hos mamma eller om man märker att barnen saknar sin pappa jättemycket, att man kan komma över en timme eller två och kanske gå ut och leka i lekparken eller någonting. Fast då måste man göra klart för barnen att “nu kommer pappa en timme eller två för att man ser att ni saknar eran pappa”(B1).)

*“C is very tricky with eating and sleeping so the routines have been there since before we separated. We talk and make sure that everything is very similar, from which songs we sing to when to sleep or routines for food*.” Mother to 2.5 year old girl quoted from: We also talk it through together if we are about to change something. C is very tricky with eating and sleeping so the routines have been there since before we separated. We talk and make sure that everything is very similar, from which songs we sing to when to sleep or routines for food. (Vi pratar alltid ihop oss om vi ska ändra någonting. C är väldigt knepig med mat och sömn så rutinerna har varit med sen innan vi separerade. Vi resonerar ihop och allting ska vara väldigt lika, från vilka sånger vi sjunger när hon ska sova till matrutiner (A19).)

”*now the 3 year old is in a period of defiance and there it is very important to have the same values. For the child rearing aspect specifically, we have to have common values*.” Father to 3 year old girl quoted from: On the other hand we have very similar opinions about child rearing. To show who is in charge. When the 3 year old is in a period of defiance and there it is very important to have the same values. For the child rearing aspect specifically, we have to have common values. Does she do this in her house I have to do likewise. Otherwise it becomes terribly wrong. It is very important that the common principles work. Like eating in front of the tv. They did it all the time when we lived together. I think that is wrong, you sit in the kitchen. There must be a special place to sit where you eat. Otherwise they can suddenly come with a cake in bed while you are sleeping. We have not entirely agreed about that (eating in front of the tv) but I cannot change X to what I think is best. (Däremot är vi väldigt lika när det gäller uppfostran, att visa vem som bestämmer. Nu är 3åringen inne i en trotsperiod och där är det väldigt viktigt att ha samma värderingar. Just på själva uppfostringsplanet så måste vi ha gemensamma värderingar. Gör hon så hos sig, så måste jag göra så hos mig annars blir det ju väldigt fel. Det är jätteviktigt att det (de gemensamma rutinerna) fungerar. Som att äta framför tvn, det gjorde dom hela tiden när vi bodde ihop. Det tycker jag är väldigt fel, man sitter i köket. Det måste finnas en speciell plats (som man äter vid) annars kan dom ju helt plötsligt komma in med en prinsesstårta i sängen när man ligger och sover. Det är vi väl inte redigt överens om (att man inte äter vid tvn) men jag kan inte förändra X till det jag tycker är bäst (B1).)

”*no, in fact I don’t think so ,[ that we have similar routines]. Because I think we’re have quite different. (…). I think children are adaptable and understand that mum does it this way and dad that way. You don’t do it the same way even if you live together*.”

Mother to a 3.5 year old boy quoted from: It is not so important that it is similar in both homes, regarding routines and so. We are fairly different. But in fact I don’t think that we have similar routines. Because I think we’re are quite different. “Ah, but I can do that at dad’s”. Yes, but you can’t do it here with me. We inform each other and try to have a similar approach to thing. I think children are adaptable and understand that mum does it this way and dad that way. You don’t do it the same way even if you live together. (Det är inte så viktigt att det ska vara "lika" på båda ställena dvs samma rutiner osv. Vi är ju ganska olika. "Ah, men såhär får jag göra hos pappa" Ja men så får du inte göra hos mig! Vi informerar varandra och försöker ha samma inställning. Vi har också samma inställning till det mesta. Jag tror barn är anpassningsbara och fattar att mamma gör så och pappa gör så. Man gör ju inte lika även om man bor ihop (A1).)

We talk a lot about routines. It doesn’t have to be similar but it is good to know about the routines in the different places (…..) We talked more about routines when she was younger. When she was little it was more routines about food but now she can say if she is hungry. I think it is really important to have common views on child rearing. But we have never needed to discuss that because we think a lot alike. So it has never been a problem. (Vi pratar mycket om rutiner. Det behöver inte vara lika men att det är bra att man känner till de olika rutinerna på de olika ställena. Vi pratade mer samma rutiner när hon var mindre. När hon var så liten så blir det mer rutiner kring mat och så men nu kan hon själv säga till om hon är hungrig. Jag tycker att det är jätteviktigt att man har samma syn på uppfostran. Men det har nog aldrig varit något vi har behövt prata så mycket om för vi tänker väldigt lika. Så det har aldrig varit något problem (A7).)

**Promoting the child-mother attachment**

*“we have said that, since [the children] are so young, they shall stay in their mother’s home. I moved to an apartment of my own (…). Practically speaking we have them half of the time, which means they sleep in my place too. But we tell them that they only live in one place, even if they also stay with me.”* Father to a 20 months old girl and a 4 year old boy quoted from: We have said that, since they are so young, they shall stay in their mother’s home. I moved to an apartment of my own and their mother stayed in our old place. Practically speaking we have them half of the time, which means they sleep in my place too. But we tell them that they only live in one place, even if they also stay with me. *(*Vi har sagt att eftersom dom är så små så ska dom bo kvar hos sin mamma. Jag flyttade till en egen lägenhet och mamma bor kvar i den gamla. I praktiken har vi dom halva tiden var, vilket innebar att dom sover hos mig också. Vi säger till dom att dom bor på ett ställe men att dom är hos mig ändå (B14).)

*”it is obvious that when X says it is not good for [the child] to be away from her more than 3 nights in a row, it is obvious that it is SHE who has problems with being away from him”*

Father to a 18 months old boy quoted from: We googled the internet, and that is one thing you often come across. It is obviously related to short memory in children. It is obvious that when X says it is not good for [the child] to be away from her more than 3 nights in a row, it is obvious that it is she who has problems with being away from him. Ha has only slept with me 4 nights in a row once without her seeing him in between. But I think 4-5 nights work but then it becomes burdensome. (Internet, vi har googlat runt och då är det oftast en sådan sak man stött på. Det ska tydligen ha att göra med att barn har kort minne. Det är tydligt på X när hon säger att det inte är bra för B att vara borta från henne mer än 3 nätter i rad så är det uppenbart att det är hon som har svårt att vara borta från honom. Han har bara sovit hos mig 4 nätter i rad en gång utan att hon inte träffat honom emellan. Men jag tycker att 4-5 nätter fungerar men sen blir det jobbigt (B18).)

*”when we moved apart he was only 7 months old and I felt it was too early and I felt that he couldn’t handle being away from me that much”* Mother to a 3 year old boy quoted from:

The most important thing with JPC is that he has an everyday contact with both of us, that both are self-evident in his life. When we separated, C was only 7 months, and I felt it was too early and I felt that he couldn't handle being away from me that much. When you pass one year and then two years, it is different but one week is still too long. Once X was gone for three weeks in a row and when he came back C was shy and didn't want to be alone with X, so I think you should have short intervals. (Det viktigaste med vvb är att han har en vardaglig kontakt med oss båda, att båda är självklara i hans liv. När vi separerade så var A bara 7 månader och då kände jag att jag inte klarade av att han skulle vara ifrån mig så mycket. När man passerar 1 år och framåt 2 år så är det skillnad men en vecka är ändå för lång tid. Som längst var X borta tre veckor i sträck och när han kom tillbaka så var B blyg och ville inte vara själv med X, så jag tror att man ska ha korta intervaller (A17).)
